# Genome sequences of Human Adenovirus 14 isolates from mild respiratory cases and a fatal pneumonia, isolated during 2006-2007 epidemics in North America

**DOI:** 10.1186/1465-9921-11-116

**Published:** 2010-08-25

**Authors:** Huo-Shu H Houng, Heping Gong, Adriana E Kajon, Morris S Jones, Robert A Kuschner, Arthur Lyons, Lisa Lott, Kuei-Hsiang Lin, David Metzgar

**Affiliations:** 1 Division of Viral Diseases, Walter Reed Army Institute of Research (WRAIR), 503 Robert Grant Avenue, Silver Spring, 20910, USA; 2 Infectious Disease Program, Lovelace Respiratory Research Institute (LRRI), 2425 Ridgecrest Dr. SE, Albuquerque, 87108, USA; 3 Clinical Investigation Facility, David Grant USAF Medical Center (DGMC), 101 Bodin Circle, Travis Air Force Base, 94535, USA; 4 Advanced Diagnostic Laboratory, Office of the Air Force Surgeon General, 2460 Pepperrell Dr, Lackland Air Force Base, 78236, USA; 5 Department of Clinical Laboratory, Kaohsiung Medical University, Shih-Chuan 1st Road, Kaohsiung,80708, Taiwan; 6 Department of Respiratory Diseases Research, Naval Health Research Center (NHRC), 140 Sylvester Rd San Diego, 92106, USA

## Abstract

**Background:**

Human adenovirus 14 (HAdV-14) is a recognized causative agent of epidemic febrile respiratory illness (FRI). Last reported in Eurasia in 1963, this virus has since been conspicuously absent in broad surveys, and was never isolated in North America despite inclusion of specific tests for this serotype in surveillance methods. In 2006 and 2007, this virus suddenly emerged in North America, causing high attack rate epidemics of FRI and, in some cases, severe pneumonias and occasional fatalities. Some outbreaks have been relatively mild, with low rates of progression beyond uncomplicated FRI, while other outbreaks have involved high rates of more serious outcomes.

**Methodology and Findings:**

In this paper we present the complete genomic sequence of this emerging pathogen, and compare genomic sequences of isolates from both mild and severe outbreaks. We also compare the genome sequences of the recent isolates with those of the prototype HAdV-14 that circulated in Eurasia 30 years ago and the closely related sequence of HAdV-11a, which has been circulating in southeast Asia.

**Conclusions:**

The data suggest that the currently circulating strain of HAdV-14 is closely related to the historically recognized prototype throughout its genome, though it does display a couple of potentially functional mutations in the fiber knob and E1A genes. There are no polymorphisms that suggest an obvious explanation for the divergence in severity between outbreak events, suggesting that differences in outcome are more likely environmental or host determined rather than viral genetics.

## Introduction

Adenoviruses are double-stranded DNA viruses. The 52 recognized serotypes of human adenovirus (HAdV) cause a broad range of symptoms: community-acquired gastrointestinal, conjunctival, and febrile respiratory illness (FRI; both upper and lower respiratory tract), hemorrhagic cystitis associated with bone marrow transplant, hepatic and urinary tract infections, and perhaps even obesity [[1-4], http://www.ncbi.nlm.nih.gov/ICTVdb/Ictv/index.htm].

The 10 serotypes of HAdV associated with FRI and pneumonia are grouped into 3 species, B (including subspecies B1 and B2), C and E, on the basis of hemagglutination and phylogenetic criteria [5-9]. HAdV-1, 2, 5, and 6, belonging to species C, cause generally endemic patterns of FRI in children and young adults [8,10,11]. In contrast, HAdV-4 (the sole serotype of species E) and the remaining respiratory species B serotypes (HAdV-3, 7, 11, 14, 16, and 21), often cause distinctive outbreaks of FRI, conjunctivitis, and pneumonia in crowded civilian populations such as dorms, public swimming pools, and boarding schools [7,8]. In the absence of vaccines, these viruses also cause almost continuous outbreaks of FRI among recruits in military training throughout the world [8,7,12,13].

Four of these seven adult human respiratory adenoviruses, HAdV-3, 7 and 21 (subspecies B1) and HAdV-4 (species E) are common, intraserotypically diverse, and inevitably represented in broad surveys [7,8,10,14]. In different populations and at different times one serotype may completely dominate this niche, several serotypes may intermingle, or multiple serotypes can appear in series through distinct replacement events [13,15-19]. The remaining three serotypes that cause FRI in healthy adults, HAdV-16 of subspecies B1 [11] and HAdV-11 and 14 of subspecies B2 [13], have only infrequently been associated with FRI. These rare associations often appear to involve more severe symptoms, outcomes, and outbreak characteristics than do those of the more common species E and subspecies B1 serotypes [11,20-24]. HAdV-14 was reported only four times in the twentieth century, always in transient, concentrated, and generally nonlethal but severely incapacitating FRI outbreaks in healthy (though crowded) adult and adolescent populations [25-28]. These outbreaks occurred between 1955 and 1963, all in Eurasia, and HAdV-14 was not reported again even in broad geographical and temporal surveys until 2001 when it was reported in 10% of FRI specimens in a retrospective analysis of clinic samples in Taiwan [29]. (Author's note: upon whole-genome analysis, this strain was identified as the very closely-related HAdV-11a; HSH, AK, data not shown). HAdV-11a has recently been seen in increased numbers of FRI cases in Asia, including some significant outbreaks [30]. Phenotypic intermediates of the closely related serotypes HAdV-11 and HAdV-14 were identified in a military camp in Spain in 1969 [31], and in Germany from a severe case of acute respiratory disease following a military training exercise (and apparently associated outbreak) in Turkey in 2004 [32].

HAdV-14 had never been identified in North America before its emergence in 2006. Following the recognized outbreaks in 2006 and 2007 [13,22,24], retrospective analysis of specific cases and collections uncovered isolated occurrences of the disease dating back a few years before the larger outbreaks (for example, see [23]). HAdV-14 was first seen in greater numbers and associated with significant outbreaks in March 2006, when it simultaneously emerged at four military recruit training centers throughout the United States, causing several hundred cases (estimated from partial surveillance) of FRI over the course of the calendar year [13]. The impact amounted to a partial replacement of the recently dominant HAdV-4, rather than an increase in overall adenoviral impact at these sites. These emergence events were not associated with symptoms or epidemiological patterns outside the normal range of those seen with the typical species E and subspecies B1 HAdVs seen in surveillance of recruit FRI and pneumonia [13].

Starting in March 2007, HAdV-14 was recognized as the cause of several severe civilian outbreaks, prompting attention from the Centers for Disease Control and Prevention (CDC) [22]. The same pathogen was recognized as the cause of prolonged outbreaks at three military installations where HAdV-14 either emerged against an adenovirus-free background ([22], and an outbreak at Coast Guard Training Center, Cape May; unpublished Naval Health Research Center [NHRC] data) or completely replaced the existing HAdV-4 strain (Marine Corps Recruit Depot [MCRD], Parris Island, NJ; unpublished NHRC data). Reported civilian HAdV-14 outbreaks were transient, lasting four months and involving nine casualties [22]. The two noted outbreaks in recruit facilities where there was no immediate history of ongoing adenovirus transmission were initially severe, involving greatly increased rates of disease among the recruits and also spreading to medical support personnel, training staff, and others. One death was reported among infected recruits as were many pneumonia hospitalizations, several requiring ventilation assistance [22].

Whole-genome restriction enzyme analysis (genome typing [33]) and partial gene sequence analysis (hexon, fiber, E1A) have shown that the currently emergent US strains of HAdV-14 (see result section for the definition of genome type HAdV-14p1), both civilian and military, are all similar at the genome type level and essentially identical (> 99.9%) at the sequence level in the hexon and fiber genes (AK, unpublished data). The circulating genome type, however, is significantly diverged from the prototype (HAdV-14p) de Wit strain (isolated from ill military recruits in the Netherlands [28]).

To further characterize the newly emergent US HAdV-14 strains, three recent HAdV-14p1 isolates were completely sequenced and compared with the prototype HAdV-14p de Wit genome sequence as well as other genetically related HAdV-11a isolates from southeast Asia. One isolate was collected in March 2006 at Marine Corps Recruit Depot, San Diego (MCRD-SD), when HAdV-14 was initially detected and identified in US recruits [13]. This isolate came from an emergence of HAdV-14p1 that did not exhibit uniquely severe outbreak dynamics or symptoms - in fact, it was observed during this outbreak, in which both HAdV-4 and HAdV-14 were present in approximately equal proportions, that HAdV-14 did not seem to cause as much pneumonia as did HAdV-4 (unpublished NHRC syndromic surveillance data). This outbreak did not involve increased rates, but rather a simple and temporary replacement of HAdV-4 with HAdV-14 [13], and the studied isolate was collected from a recruit with uncomplicated FRI. The other two sequenced HAdV-14 isolates were collected at Lackland Air Force Base during the severe and prolonged outbreak of HAdV-14 that started in February 2007 and drew the attention of the CDC a month later, as the outbreak spread [22]. One was from a fatal case of respiratory failure from viral pneumonia, which followed several weeks of intubation and life support. The other was from a mild case of FRI.

The primary goal of our study was to determine if there were any apparent genetic correlates that might distinguish viruses causing mild and severe outbreaks or mild and severe symptoms, or differences between the currently circulating strain of HAdV-14p1 and the prototype HAdV-14p that was seen in Eurasia in the 1950 s and 1960 s. A secondary goal was to identify unique signature sequences that might allow us to track individual strains of HAdV-14 of US origin for the purposes of epidemiological investigations.

## Materials and methods

### Sample Collection

The isolate from a mild FRI outbreak at MCRD San Diego was collected with consent under an institutional review board-approved research protocol (NHRC.1999.0002), identified, cultured, and analyzed as part of NHRC's ongoing population-based FRI surveillance program. The two isolates from Lackland Air Force Base included one NHRC surveillance isolate from a recruit with uncomplicated FRI and one fatal pneumonia isolate collected from a severely ill recruit at Wilford Hall Medical Center, initially collected for diagnostic viral culture and later provided to LRRI and WRAIR as a de-identified isolate.

NHRC samples were collected as oropharyngeal (throat) swabs in VTM (Remel, Lenexa, KS), immediately frozen in either -80°C freezers or on dry ice, and transported on dry ice to NHRC under College of American Pathologists (CAP)-accredited collection and transport protocols. Lackland Air Force Base samples were collected as throat swabs in VTM, cultured in A549 cells, and transported to NHRC as above. All samples were tested at NHRC for HAdV-14 [13] as raw specimens, then subsequently cultured in A549 cells (Diagnostic Hybrids Inc., Athens, OH), and stored frozen as infected tissue culture fluid (isolated virus) at Lovelace Respiratory Research Institute (LRRI). Sequencing work on these samples was performed at Walter Reed Army Institute of Research (WRAIR) on the resulting isolates.

### Sanger Sequencing of HAdV-14

PCR and sequencing were accomplished at the virology facility at Walter Reed Army Institute of Research (WRAIR), Silver Spring, Maryland, USA. PCR primer pairs were designed from the prototype HAdV-14p de Wit sequence (GenBank accession number AY803294) and used to generate overlapping 1-2 kilobase amplicons covering the entire genome. All PCR products were sequenced in both directions by using forward and reverse PCR primers corresponding to each individual PCR product. All clean and verified readable sequences were used to assemble full HAdV-14 genome sequences using the Sequencher software (Gene Codes Corporation, Ann Arbor, MI).

Two hundred microliter aliquots of each isolate were extracted using the Invitrogen ChargeSwitch DNA extraction kit (Invitrogen Corporation, Carlsbad, CA) per the manufacturer's instructions, and resuspended in 200 μl elution buffer. One hundred microliter PCR amplification reactions consisted of 2 mM MgCl2, 0.6 mM dNTP (1.5 mM each A, C, T, and G), 200 μM each primer, 2.5 units Platinum Taq Polymerase (Invitrogen), and 1 ul of extracted isolate in 1X ABI Buffer II (Applied Biosystems Inc., Foster City, CA). Thermal cycling was carried out on an ABI9700 platform (Applied Biosystems) using the following parameters: initial activation for 2 min at 94°C, then 35 cycles of: 20 s at 94°C, 20 s at 53°C, and 2 min at 72°C. Final extension was for 7 min at 72°C. PCR cleanup was performed using the Qiagen PCR cleanup kit (Qiagen, Valencia, CA) per the manufacturer's instructions. Sequencing reactions were set up per the manufacturer's instructions using the ABI BigDye Terminator kit (manual version 3.2, Applied Biosystems), and run on an ABI9700 platform. Reaction products were analyzed on an ABI3130XL automated sequencer (Applied Biosystems) per the manufacturer's instructions. Resulting data were then edited and aligned using Sequencher software (Gene Codes).

## Results and Discussions

### HAdV-14 Genomes from 2006-7 US Outbreaks

**Figure 1:**
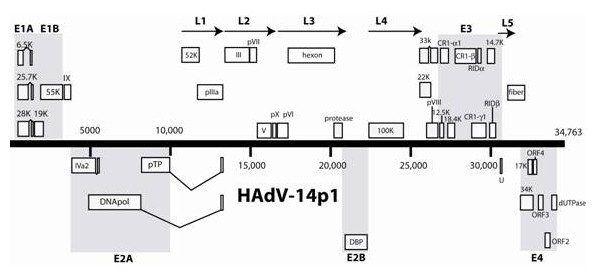
**Map of apparent open reading frames and their identities in the genome of the emerging North American HAdV-14p1**.

Genomic sequences from three different recent US HAdV-14 isolates, including two from Lackland Air Force Base (303600 and 1986T, associated with mild FRI and fatal pneumonia, respectively, both from a severe outbreak) and one from Marine Corps Recruit Depot, San Diego (NHRC22039, associated with a mild infection during a mild outbreak) were fully sequenced, assembled and submitted to the NCBI GenBank database (GenBank Accession #s FJ822614, EU827616 and EU833993, respectively). The genome size of Lackland strains 303600 and 1986T is identical to each other, 34,764 base pairs (bp) that is also identical in size with the HAdV-14 prototype deWit strain, 34,764 base pairs (bp). The San Diego strain NHRC22039 has a genome size of 34,768 bp. All three recent US HAdV-14 strains are highly homologous with each other. The two Lackland strains are 100% identical to each other, while the San Diego isolate differed only by a 4 bp extension of the polyadenylation signal (a poly-T on the coding strand) at the 5' end of the terminal binding protein (TBP) gene, and a single noncoding (synonymous) base substitution in the fiber gene. The poly-T repeat is 13 bp long [T(13)] in the Lackland strains and T(17) in the San Diego strain. HAdV-14p strain deWit contains a corresponding T(11) repeat. The genomes of all three recent US HAdV-14 isolates share identical coding regions for all genes. As recently described, and after detailed characterization by restriction enzyme analysis, all North American isolates of HAdV-14 correspond to genome type 14p1 [34]. Table 1 shows the summary of alignment results. HAdV-14 deWit and the recent US HAdV-14p1 isolates differ by 0.3%, scattered quite evenly through the genome. All 4 HAdV-14 s examined in this study have the same base composition of 51.2% A/T, 48.8% G/C. The GC content and the number of open reading frames (ORFs) were identical to the HAdV-14p de Wit strain. A map of the organization of predicted ORFs within the genome of the emerging HAdV-14p1 strain is shown in Figure 1, and is identical to that of the prototype HAdV-14 de Wit strain.

**Table 1:** Summary of Alignment Results

	deWit	NHRC	30600	1986T
deWit	100.0	99.7	99.7	99.7

NHRC	99.7	100.0	100.0	100.0

30600	99.7	100.0	100.0	100.0

1986T	99.7	100.0	100.0	100.0

All HAdV genomes are bounded by inverted terminal repeats (ITR) ranging from 100 to 200 bp in size, which serve as viral replication origins. The representative ITRs of various HAdV species, such as species A (HAdV-12, 18, 31), B (HAdV-3, 7, 11), and C (HAdV-1, 2, 5) are available in GenBank. Among these HAdVs, ITRs are highly conserved within species but diverse between species. For example, the ITRs of HAdV-2 and HAdV-5 (species C) are identical 103 bp sequences. Similarly conserved ITR patterns are observed for the 137 bp ITRs of species B (HAdV-3, 7 and 11). However, all three recent US HAdV-14 s share identical inverted terminal repeat (ITR) sequence of 133 bp, in contrast to other species B HAdVs. The HAdV-14 prototype deWit also contains a 133 bp ITR, but this differs from recent HAdV-14 s of US origin by one substitution at base pair 68 (T68C). This level of polymorphism in ITR sequences among closely related strains is unusual.

**Figure 2:**
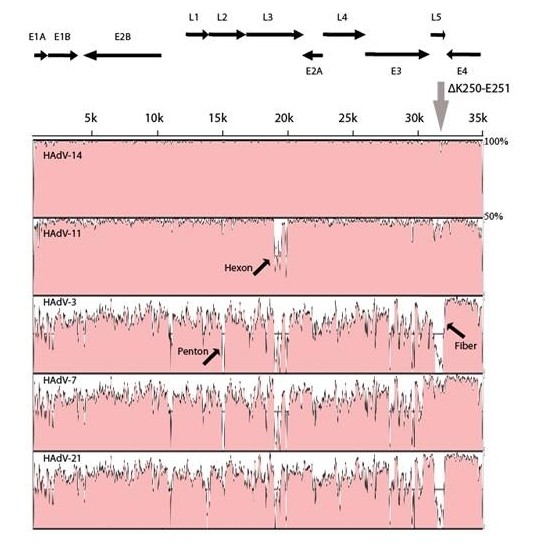
**Global pairwise comparison of multiple species B HAdV genomes**.

We compared HAdV-14p1 to selected HAdV-B prototype strains (HAdV-14, 3, 7, 11, and 21) using the mVISTA Limited Area Global Alignment of Nucleotides (LAGAN) tool (MontaVista Software, Inc., Santa Clara, CA) [35] (Figure 2). With the exception of the hexon gene of HAdV-11p, HAdV-14p1 showed strong homology with both HAdV-14p and -11p. This was expected, since HAdV-11 and HAdV-14 are both members of subspecies B2. Comparison of HAdV-14p1 to HAdV-3, 7, and 21, members of subspecies B1, revealed sequence divergence throughout the genome, especially the penton, hexon, and fiber genes (Figure 2). These data are consistent with serological identification of the new strain as HAdV-14, since the hexon in the primary antigenic determinant and the penton and fiber act as secondary antigenic determinants.

**Figure 3:**
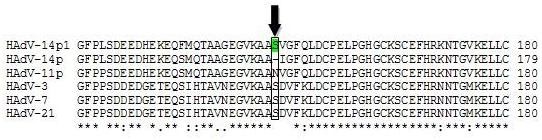
**E1A alignments**. Alignment of selected E1A 28K amino acid sequences from HAdV-3, 7, 11, 14p, 14p1, and 21. Black arrow demarcates the S147 insertion, shared by HAdV-3, 7, and 21.

**Figure 4:**
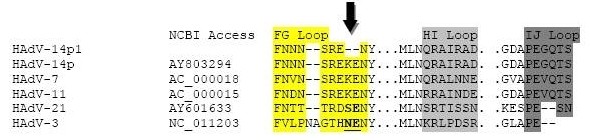
**Fiber knob binding site alignment**. Alignment of the amino acid sequences on the exposed regions of the fiber loops FG, HI, and IJ that are involved in binding to CD46. Black arrow demarcates the ΔK250-E251 deletion. All sequences were aligned using the ClustalX [40] alignment method. The following HAdV genomes (GenBank accession numbers) were used: HAdV-14p (AY803294), HAdV-11p (AY163756), HAdV-3 (NC_011203), HAdV-7 (AC_000018), and HAdV-21 (AY601633).

The nucleotide identity scores for HAdV-14p1 genes with less than 100% identity with HAdV-14p are shown in Table 2. There were 19 nucleotide polymorphisms observed. Only two of these affected amino acid coding sequences. The first was a 3-bp insertion in the HAdV-14p1 sequence, which resulted in an inserted serine at position #147 of the 25.7 K and 28 K protein sequences (Figure 3). The proteins encoded by E1A regulate the transcription of viral as well as cellular genes [36,37]. The second was a 6-bp deletion in the fiber gene of the HAdV-14p1 sequence. This resulted in a two amino acid deletion in the FG loop of the fiber gene (Figure 4). The fiber gene is responsible for mediating attachment of the adenovirus to the host cell [38,39].

**Table 2:** Percent identities of the nucleotide coding sequences of selected HAdV-14p1 genes to homologous sequences of the HAdV-14p and HAdV-11p.

Protein	% nucleotide identity	
	HAdV-14p	HAdV-11p
E1A 28K	98	96
E1A 25.7K	97	97
IVa2	99	98
DNA Polymerase	99	99
pTP	99	99
Penton	99	97
Hexon	99	92
100K	99	98
22K	99	97
CR1-α1	98	95
CR1-β1	99	94
CR1-γ1	98	91
RIDβ	98	95
Fiber	99	98
ORF6/7	97	97
ORF1	98	95

Although at the nucleotide level the genomes of HAdV-14p de Wit and HAdV-14p1 strains were highly homologous, we wanted to determine whether there was evidence of recombination. SimPlot bootscan analysis of HAdV-14p1 with respect to prototypical strains of HAdV-3, 4, 7, 11, 14, and 21 demonstrated that this virus is mostly closely related to the HAdV-14p prototype strain and is not a recombinant with respect to other recognized serotypic clades (data not shown). As noted previously, polymorphisms between these two strains were distributed evenly throughout the genome.

The three sequenced strains of HAdV-14p1 were almost identical. The two Lackland isolates were exactly the same, while the San Diego strain differed by the addition of four extra Ts to the TBP polyadenylation signal repeat, and by a single synonymous base substitution in the fiber gene.

## Conclusions

HAdV-14p1 (strain 303600) appears to be a closely related direct drift variant of the HAdV-14p (strain de Wit) prototype seen in the past, differing primarily by an insertion in E1A, a small deletion in the fiber gene, and a few other coding single nucleotide polymorphisms (SNPs) in E3 and other genes. The deletion (ΔK250-E251) in the fiber gene is the most notable genetic difference between the HAdV-14p and HAdV-14p1 (Figure 4). Despite the observed ΔK250-E251 deletion, the fiber gene sequence of HAdV-14p1 shares greater overall homology with the fiber of HAdV-11a than that of HAdV-11p. Whereas, HAdV-11p causes mostly urinary tract infections and shares very low fiber homology with HAdV-11p1, HAdV-11a and HAdV-14p all causing respiratory infections [34]. This is consistent with the receptor-binding role of the fiber and the close relationship between receptor specificity and organ tropism. HAdV-14p1 and HAdV-11a both cause upper respiratory infections, while HAdV-11p causes mostly urinary tract and bone marrow infections in transplant patients. Species B viruses are unique in that they use CD46, a complement protein, as a receptor [38]. Many other human adenoviruses use the CAR protein [38]. The deleted amino acids could affect the exposed region of the FG loop by altering the overall affinity for CD46. A less likely alternative is that the fiber deletion influences an interaction with a receptor other than CD46, such as CAR. A third possibility is that this deletion has no affect at all on the fiber gene. Whether this mutation affects the pathogenicity of HAdV-14p1 compared with HAdV-14p will require further studies.

When HAdV-14p was first identified in Eurasia in the 1950 s and 1960 s, it generated localized, high attack rate epidemics of FRI similar to those seen with the current strain. After a decade of sporadic activity, it disappeared and remained almost completely undetected for 4 decades. As a recently emerged virus, HAdV-14p1 has an increased potential for high rates of transmission and high attack rates, simply because the vast majority of North Americans are likely to have never been exposed (essentially the entire population is susceptible). This is similar to the situation long recognized for HAdV-4, which, in the absence of vaccines, has always been the dominant serotype affecting military recruits. Serosurveys have generally indicated that a greater proportion of the young adult population is susceptible to HAdV-4 than to other common respiratory serotypes such as HAdV-7.

The two sequenced strains from Lackland Air Force Base, one from a severe (fatal) pneumonia and one from a mild case of acute respiratory disease, were identical. There were only two noncoding polymorphisms distinguishing the Lackland isolates from the San Diego isolate (another mild case). The poly-T length polymorphism was studied in a wide range of isolates from multiple sites, and found to be a hypervariable and useful source of geographically specific strain identity information [40]. Neither mutation suggested a significant genetic source of variation in clinical severity. The results supported previous observations of a high degree of conservation in hexon and fiber genes relative to the prototype HAdV-14p and to the closely related HAdV-11a.

## Competing interests

The authors declare that they have no competing interests.

## Authors' contributions

HG carried out the sequencing of Ad14 genomes. AEK, MSJ, RAK, AL, LL, KL and DM all participated in the samples collections, sequencing alignment and draft of manuscript. All authors read and approved the final manuscript submission.
